# Artificial intelligence in endoscopic prediction of submucosal invasion in gastric neoplasms: systematic review and meta-analysis

**DOI:** 10.1016/j.clinsp.2026.101010

**Published:** 2026-06-24

**Authors:** Dan Long, Jinrong Zeng, Qiu Jianting

**Affiliations:** Department of Gastroenterology, The Second Hospital of Longyan, Longyan Fujian, China

**Keywords:** Artificial intelligence, Endoscopy, Gastric neoplasms, Submucosal invasion, Diagnostic accuracy

## Abstract

**Background:**

Accurate assessment of Submucosal (SM) invasion in gastric neoplasms is vital for curative endoscopic management, yet conventional endoscopy remains moderately accurate and operator dependent. Whether AI can deliver reliable, generalizable depth prediction is unknown.

**Aim:**

This investigation establishes the diagnostic capability of AI-driven endoscopic systems for identifying SM invasion. Methods: A PRISMA-DTA systematic review and meta-analysis identified studies that used machine-learning or deep-learning models applied to endoscopic imaging with histopathology as the reference standard. Validation-set 2 × 2 data were pooled using hierarchical models to estimate sensitivity, specificity, and AUC, and heterogeneity was assessed through subgroup analyses.

**Results:**

Eight studies were included in the systematic review; seven studies provided sufficient data (including ten cohorts for the meta-analysis). AI systems demonstrated a pooled sensitivity of 0.75 (95% CI 0.69–0.81) and a specificity of 0.84 (95% CI 0.79–0.87). Discriminatory ability was high (AUC = 0.87, 95% CI 0.84–0.90), providing a conservative, transportable estimate of performance based on validation cohorts. Heterogeneity was substantial. Externally validated models showed lower sensitivity than internally validated models (0.72 vs. 0.79; p < 0.001), revealing overfitting. Korean cohorts demonstrated higher specificity than studies from other regions (0.87 vs. 0.79; p < 0.001). Fagan's analysis showed that a positive AI result increased post-test probability from 25% to 61%, while a negative result reduced it to 9%.

**Conclusions:**

AI-assisted endoscopic assessment establishes a high-accuracy framework for identifying SM invasion and opens the door to more consistent treatment selection in early gastric cancer. International external validation and unified invasion definitions remain essential for dependable clinical use.

## Highlights

• AI endoscopy predicts submucosal invasion with high accuracy in gastric neoplasms.
• 10 validation cohorts pooled: sensitivity 0.75 and specificity 0.84.
• Discrimination was strong (AUC = 0.87), but heterogeneity was substantial.
• External validation reduced sensitivity, highlighting overfitting risk in AI models.
• Clinical utility: post-test probability rose to 61% after a positive AI prediction.

## Introduction

Gastric cancer remains a major global health burden, ranking among the leading causes of cancer-related mortality despite declining incidence in some regions. Early Gastric Cancer (EGC), defined as a tumor confined to the mucosa or submucosa regardless of lymph node status, carries an excellent prognosis when appropriately treated, making accurate staging of invasion depth clinically pivotal. Submucosal (SM) invasion beyond 500 µm (T1b2) substantially increases the risk of lymph node metastasis and shifts management from endoscopic therapy toward gastrectomy with lymphadenectomy. Consequently, reliable pre‑treatment assessment of SM involvement is central to selecting optimal, organ‑preserving strategies for gastric neoplasms.^1^^,^^2^

Endoscopic Submucosal Dissection (ESD) has become an established curative option for selected superficial gastric neoplasms, enabling resection as a single piece with low local recurrence and acceptable complication profiles. Guideline-based indications for ESD rely heavily on predictions of tumor size, histology, ulceration, and especially depth of invasion, which determine the risk of non-curative resection and the need for additional surgery. However, conventional White-Light Endoscopy (WLE) and Endoscopic Ultrasound (EUS) have only moderate and variable accuracy for differentiating Mucosal (M) from SM disease, with reported EUS accuracy for EGC depth ranging from approximately 56% to 88%. This diagnostic uncertainty may lead to both overtreatment with unnecessary surgery and under-treatment with incomplete endoscopic resection.^3^, ^4^, ^5^

Despite these technological and diagnostic limitations, accurate endoscopic diagnosis also depends heavily on the operator. Substantial skill and familiarity with subtle mucosal features are required, yet even with extensive training, human limitations and equipment constraints can result in overlooked abnormalities or misclassification. Prior studies have shown that diagnostic accuracy is positively correlated with the endoscopist’s level of experience.^6^, ^7^, ^8^

Artificial Intelligence (AI), particularly Deep Learning (DL) and Convolutional Neural Networks (CNNs), has rapidly emerged as a transformative tool in gastrointestinal endoscopy. In upper gastrointestinal imaging, AI systems now assist in lesion detection, characterization, and risk stratification, aiming to reduce operator dependence and interobserver variability. For EGC, CNN‑based algorithms have achieved high sensitivity and specificity for lesion detection and classification, performing comparably or superior to expert endoscopists while operating in near real time. These advances have spurred intense interest in extending AI applications beyond mere detection to clinically meaningful tasks, such as predicting invasion depth and resection curability.^9^, ^10^, ^11^

Recent work has specifically targeted AI‑based estimation of SM invasion in gastric neoplasms from endoscopic images and multimodal inputs. CNN models trained on magnifying endoscopy with narrow‑band imaging have shown a promising ability to discriminate M from SM invasive cancers based on microvascular and microsurface patterns, with reasonable accuracy for depth prediction. Beyond pure image analysis, newer machine‑learning frameworks integrate endoscopic features with clinical and imaging variables, including standardized colorimetric metrics and EUS findings, to improve the classification of deep SM invasion in EGC. These methods could be embedded as real‑time decision support tools, potentially standardizing assessment across centers and experience levels.^12^^,^^13^

Despite growing interest, the evidence base for AI‑assisted prediction of SM invasion in gastric neoplasms remains heterogeneous, encompassing diverse model architectures, imaging modalities, outcome definitions, and validation strategies. Existing narrative and umbrella reviews on AI in digestive endoscopy have largely focused on neoplasia detection and overall diagnostic performance, without quantitatively synthesizing accuracy specifically for invasion‑depth prediction in gastric lesions. Furthermore, the generalizability of reported models across populations, devices, and practice settings is uncertain, and the relative performance of AI compared with expert endoscopists for this task is not clearly defined.^10^^,^^11^^,^^14^

Therefore, a dedicated systematic review and meta‑analysis are needed to rigorously evaluate the diagnostic performance of AI‑based systems for endoscopic prediction of SM invasion in gastric neoplasms, identify sources of heterogeneity, and clarify their potential role in guiding treatment selection.

## Methods and materials

The conduct and reporting of this systematic review and meta-analysis followed the PRISMA-DTA recommendations for studies evaluating diagnostic test accuracy.^15^ The Ncompleted PRISMA-DTA checklist is provided in Supplementary Material S1.

### Search strategy

To identify all eligible studies, two reviewers independently performed a systematic and comprehensive search of PubMed, EMBASE, and Web of Science from database inception through 10 November 2025. The search strategy combined controlled vocabulary and free-text terms related to AI and endoscopic assessment of gastric neoplasms. The following keywords and Boolean operators were applied: (“*artificial intelligence*” *OR AI OR* “*machine learning*” *OR ML OR “deep learning*” *OR DL OR* “*neural networks*” *OR* “*convolutional neural networks*” *OR CNN) AND (endoscopy OR endoscopic OR gastroscopy) AND (submucosal OR mucosal invasion) AND* (“*gastric cancer*” *OR* “*stomach cancer*” *OR* “*gastric carcinoma*” *OR* “*stomach neoplasm*”). All retrieved citations were imported into Rayyan, an online platform designed to facilitate systematic review management and minimize the risk of missing relevant literature.^16^ Duplicate records were automatically and manually removed before screening. Subsequently, two reviewers independently assessed titles and abstracts, excluding articles that clearly failed to meet the predefined inclusion criteria. Studies considered potentially relevant were evaluated in full text. Any disagreements between reviewers were resolved through discussion, and when necessary, by consulting a third reviewer to reach a consensus.

### Inclusion and exclusion criteria

A predefined PICO framework was used to guide the scope and selection of studies. The review focused on adult patients with gastric neoplasms undergoing endoscopic evaluation (Population), in whom AI-based methods, including Machine Learning (ML) and DL algorithms, were applied to endoscopic imaging to predict SM invasion (Intervention). Eligible studies compared AI-derived predictions against established diagnostic standards, such as histopathological assessment or expert endoscopist interpretation (Comparison). They reported diagnostic performance outcomes, including sensitivity, specificity, accuracy, and the area under the receiver operating characteristic curve (Outcome). To maintain clinical and methodological homogeneity for decision-making around endoscopic resection, the authors limited eligible index tests to conventional optical endoscopy (white-light endoscopy with or without image-enhanced modalities such as NBI/LCI/BLI) and excluded EUS-only AI systems and other non-optical modalities. In addition, the target condition was defined as a binary prediction of SM invasion versus mucosa-confined disease (T1b/SM+ vs. T1a/M or equivalent thresholds), enabling construction of 2 × 2 diagnostic tables for meta-analysis.

In alignment with this framework, studies were considered eligible if they investigated AI-based analysis of endoscopic images for the assessment of SM invasion in gastric neoplasms, provided sufficient methodological detail to allow appraisal of the AI model, and reported quantitative performance metrics. Only peer-reviewed articles published in English were included. Studies were excluded if they did not involve AI-driven evaluation of endoscopic imaging, did not address the prediction of SM invasion, relied solely on non-endoscopic imaging modalities, lacked adequate methodological transparency, or were published as non-original research, including case reports, reviews, conference abstracts, book chapters, comments, or letters.

### Data extraction

Two reviewers independently performed data extraction to maintain consistency and reduce potential errors. A standardized Google Sheets template was used to record all relevant information systematically. For each study, the authors gathered key publication and methodological details, including the first author, publication year, country, study design (prospective or retrospective), and whether the investigation was conducted at a single center or across multiple centers. Clinical characteristics were also collected, including the specific type of gastric neoplasm assessed, the reference standard used to confirm SM invasion, and the definition of SM invasion adopted by each study. When available, the authors extracted the number of patients or images included and the number of endoscopists involved, particularly in studies comparing diagnostic performance between clinicians and AI systems.

Imaging-related characteristics were systematically documented, including the imaging modality, image type, and any relevant technical details. Information on AI methodologies was also compiled, including whether studies employed ML or DL approaches, the algorithmic models used, and the software or programming platforms used for model development.

### Quality assessment

The methodological rigor of the included studies was appraised using a customized version of the Quality Assessment of Diagnostic Accuracy Studies-2 (QUADAS-2) tool. QUADAS-2 evaluates the potential for bias within four key domains, including patient selection, index test, reference standard, and flow and timing, and examines concerns regarding applicability in the first three domains. To ensure the tool reflected the specific methodological considerations of AI-driven endoscopic diagnostics, the authors adapted the original signaling questions to account for the characteristics and challenges inherent to AI-based research.^17^ These modifications allowed for a structured evaluation of study design, data management, model training and validation, and the clinical relevance of AI-generated predictions across each domain. The complete set of adapted signaling questions used in this review is available in the Supplementary Materials S2 to support transparency and reproducibility.

### Meta-analysis

The meta-analysis was carried out using STATA version 15, employing the MIDAS module to synthesize diagnostic test accuracy outcomes. Pooled sensitivity, specificity, and their corresponding 95% Confidence Intervals were calculated to summarize the overall diagnostic performance of AI systems for predicting SM invasion in gastric neoplasms. A Summary Receiver Operating Characteristic (SROC) curve was developed, and the Area Under the Curve (AUC) was computed to represent the collective discriminatory ability of the included models. To complement these analyses, forest plots were produced to visually depict the distribution of sensitivity and specificity across individual studies.

Heterogeneity was examined using Cochran’s *Q*-test and the *I*^2^ statistic, with *I*^2^ values categorized as very low (0%–25%), low (26%–50%), moderate (51%–75%), or high (> 75%). To further explore potential contributors to between-study variability, predefined subgroup analyses were performed. These analyses compared diagnostic performance across multiple study characteristics, including publication period (studies conducted before 2024 versus those published thereafter), type of validation strategy (internal compared with external validation), country of origin (Korea versus other regions), variation in the operational definition of SM invasion, and the software environment used for constructing the AI models, particularly contrasting Python-based platforms with other analytical tools. These comparisons were undertaken to identify methodological or contextual factors that might influence accuracy estimates. These subgroup analyses were a priori hypothesis-generating, intended to contextualize pooled estimates and identify features most likely to influence model transportability. Given the limited number of available validation cohorts, subgroup analyses were interpreted as exploratory and hypothesis-generating rather than definitive tests of effect modification.

The possibility of publication bias was assessed using Deeks’ funnel plot asymmetry test. Additionally, the clinical utility of the AI-derived models was explored using Fagan’s nomogram to estimate post-test probabilities under varying pre-test probability assumptions, thereby illustrating the potential impact of these models on real-world clinical decision-making.

## Results

### Study selection

**Fig. 1:**
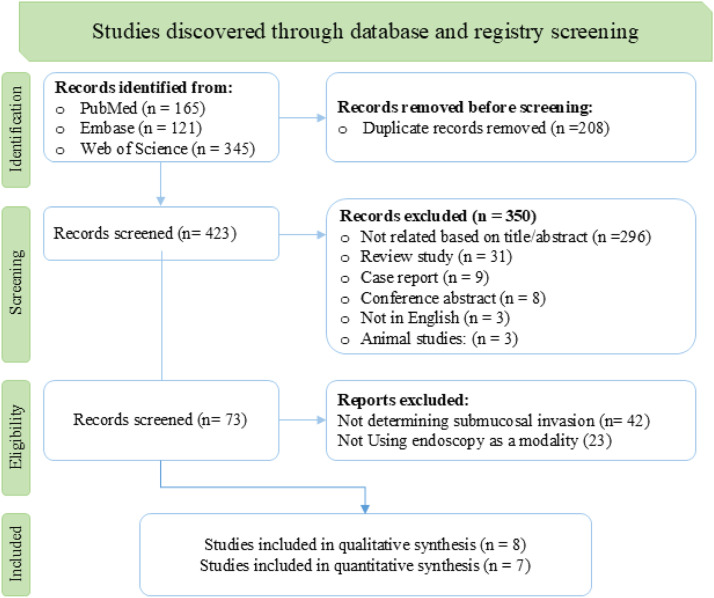
PRISMA flow diagram of study selection.

The database search yielded 631 citations. After eliminating 208 duplicate records, 423 unique studies proceeded to title and abstract screening. During this stage, 350 records were excluded due to factors such as lack of relevance to the research topic, animal-based investigations, reviews, case reports, conference abstracts, or non-English publications. Consequently, 73 full-text articles were examined for eligibility. Based on the predefined inclusion and exclusion standards, 65 studies were subsequently removed after full-text assessment, with specific exclusion reasons outlined in Fig. 1 (PRISMA flow diagram). In the end, eight studies met all criteria and were included in the systematic review.^18^, ^19^, ^20^, ^21^, ^22^, ^23^, ^24^, ^25^ Of these, seven studies provided sufficient validation-set data to reconstruct 2 × 2 tables and were therefore included in the quantitative meta-analysis, contributing ten validation cohorts.

The smaller number of included depth-assessment studies compared with broader AI-EGC reviews reflects the a priori restriction to optical endoscopy-based binary SM invasion tasks and the requirement for extractable validation-set data.

### Quality assessment

**Fig. 2:**
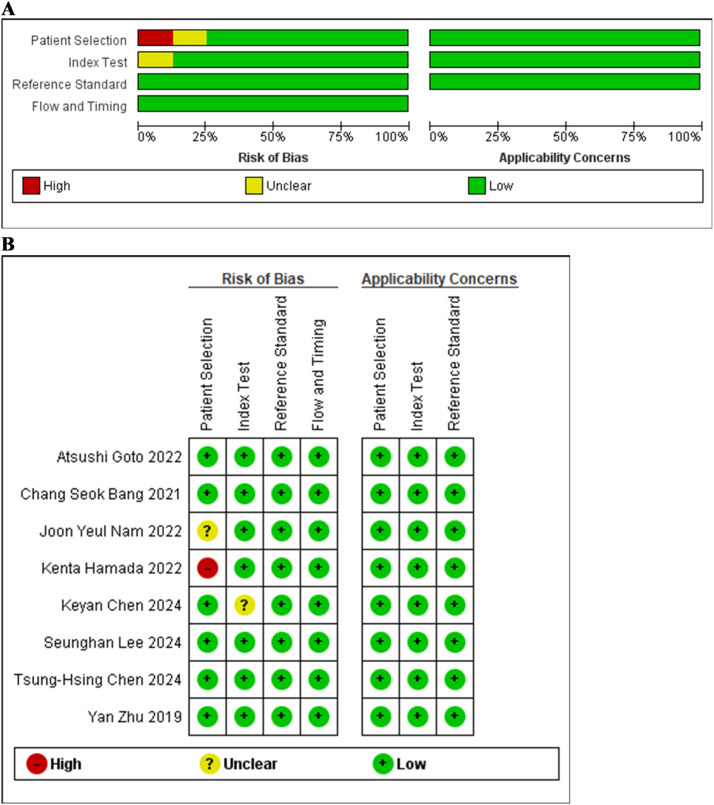
(a) Summary of QUADAS-2 risk of bias and applicability assessment. (b) QUADAS-2 domain-level ratings for each included study.

The methodological quality of the included studies was generally satisfactory, as reflected in the QUADAS-2 assessment (Fig. 2a and 2b). Across all investigations, concerns about applicability were consistently rated low, indicating that the study populations, diagnostic approaches, and reference standards were well aligned with the aims of this review.

With respect to risk of bias, most studies demonstrated low risk across all domains. Nonetheless, a few exceptions were noted. One study was judged to have a high risk of bias in patient selection, largely due to its retrospective, single-center design and the deliberate balancing of lesion categories, which may have altered the natural case distribution.^23^ Another study showed an unclear risk in the selection domain, as only a single image per patient was manually chosen by an expert reviewer, potentially limiting representativeness and introducing subjective selection tendencies.^22^ Additionally, one study reported an unclear risk in the index test domain, in which endoscopic feature extraction was performed retrospectively by experienced clinicians, potentially biasing interpretations by knowledge of histologic outcomes.^21^ Apart from these issues, the remaining studies maintained low risk across the evaluated criteria.

### General extracted data

Table 1 summarizes the key characteristics of the eight studies included in this review, which were published between 2019 and 2024. The studies were geographically concentrated in East Asia, with three conducted in the Republic of Korea, two in Japan, two in China, and one in Taiwan.

**Table 1:** Characteristics of included studies.

First author	Year	Country	Design	Centers (N)	Reference Standard	Submucosal Invasion Definition	Patients / Images (N)	Image modality	Image type	AI model	Algorithm	Software
Seunghan Lee	2024	Korea	R + P	Multicenter (2)	Postoperative histopathologic	Submucosal invasion < 500 µm = SM1; ≥ 500 µm = SM2	5032 images; 2159 patients; 252 videos	White-light endoscopy	Still images videos	DL	CNN	Pytorch
Atsushi Goto	2022	Japan	R	Multicenter (2)	Postoperative histopathologic	Submucosal invasion < 500 µm = SM1; ≥ 500 µm = SM2	500 images	white-light endoscopy	Still images	DL	CNN	Python
Tsung-Hsing Chen	2024	Taiwan	R	Multicenter (2)	Postoperative histopathologic	pathologic T category (T1a = mucosal, T1b = submucosal)	893 images	White-light endoscopy Narrow-band imaging	Still images	DL	CNN	Python
Keyan Chen	2024	China	R	Single center	Postoperative histopathologic	Histopathologic classification as M vs. SM	718 patients	White-light endoscopy	Still images	ML	LR and RF	Python
Joon Yeul Nam	2022	Korea	R	Multicenter (2)	Postoperative histopathologic	pathologic T category (T1a = mucosal, T1b = submucosal)	1366 patients	White-light endoscopy	Still images	DL	CNN	Tensor Flow
Kenta Hamada	2022	Japan	R	Single center	Postoperative histopathologic	Submucosal invasion < 500 µm = SM1; ≥ 500 µm = SM2	200 patients; 3508 images	White-light imaging. Linked color imaging. Blue laser imaging. Indigo carmine dye contrast imaging	Still images	DL	CNN	Chainer
Chang Seok Bang	2021	Korea	R + P	Multicenter (3)	Postoperative histopathologic	pathologic T category (T1a = mucosal, T1b = submucosal)	2899 images	White-light endoscopy	Still images	DL	CNN	Neuro-T
Yan Zhu	2019	China	R	Single center	Postoperative histopathologic	Submucosal invasion < 500 µm = SM1; ≥ 500 µm = SM2	993 images	White-light endoscopy	Still images	DL	CNN	Python

N, Number; R, Retrospective; P, Prospective; AI, Artificial Intelligence; DL, Deep Learning; ML, Machine Learning; CNN, Convolutional Neural Networks; RF, Random Forest; LR, Logistic Regression.

All investigations employed a retrospective study design, although two supplemented this with a prospective component.^18^^,^^24^ In terms of study setting, five studies were conducted across multiple centers, and three were single-center.^21^^,^^23^^,^^25^ Among the multicenter investigations, one incorporated data from three institutions,^24^ while the remaining four involved two centers each.

Across all studies, postoperative histopathological assessment following ESD or surgical resection was used as the reference standard. The definition of SM invasion varied across the included studies, with most distinguishing invasion depth using a micrometer threshold (commonly < 500 µm vs. ≥ 500 µm). Other studies classified invasion based on pathologic T categories (T1a vs. T1b) or histopathologic differentiation between M and SM carcinoma. One study further used a binary system distinguishing endoscopically resectable lesions (≤ SM1) from non-resectable lesions (> SM1). The specific criteria applied in each study are detailed in Table 1.

WLE served as the primary imaging modality in all included studies. One study also integrated narrow-band imaging,^20^ and another expanded its imaging spectrum by adding Linked Color Imaging (LCI), Blue Laser Imaging (BLI-bright), and non-magnifying indigo carmine dye contrast imaging.^23^ Still images were used across all datasets, although one study additionally employed video sequences.^18^

Regarding AI methodology, seven studies implemented DL models, predominantly based on CNNs. The remaining study utilized an ML approach incorporating random forest and logistic regression algorithms.^21^

Most investigations reported using Python as the main software environment for model development and analysis.

### Meta-analysis

#### 2 × 2 construction

For each eligible study, the authors reconstructed diagnostic 2 × 2 contingency tables comprising True Positives (TP), False Positives (FP), True Negatives (TN), and False Negatives (FN) using the information reported for the corresponding validation cohorts. Of the eight studies included in the review, one lacked adequate data to generate a complete contingency table,^25^ leaving seven studies that collectively contributed ten validation cohorts (both internal and external) to the quantitative synthesis.

In most studies, TP indicated lesions correctly identified as having SM invasion, and TN indicated lesions correctly classified as mucosa-confined, with FP and FN defined in the conventional way. Two studies, however, adopted an inverse labeling system, differing from the standard diagnostic convention.^20^^,^^23^ For these, contingency values were manually recalculated to ensure consistency across all datasets before pooling.

Notably, only validation-set data were used in the meta-analysis; performance metrics derived from training sets were deliberately excluded to minimize the risk of overfitting and to maintain the objectivity and generalizability of the aggregated estimates. This design choice may yield lower pooled sensitivity than analyses that incorporate training or internally optimized results, but it provides a more transportable estimate for clinical use. The finalized contingency tables used for quantitative analysis are summarized in Table 2.

**Table 2:** Constructed 2 × 2 table.

Study	Data set	Total number	SM number	M number	Sensitivity	Specificity	TP	FN	TN	FP
Seunghan Lee	Internal validation	110	56	54	0.833	0.911	45	9	51	5
Seunghan Lee	External validation	242	77	165	0.533	0.806	41	36	133	32
Atsushi Goto	External validation	200	100	100	0.74	0.71	74	26	71	29
Tsung-Hsing Chen	Internal validation	893	351	542	0.809	0.77	281	70	418	124
Tsung-Hsing Chen	External validation	56	9	47	0.829	0.777	39	8	7	2
Keyan Chen	Internal validation	214	57	157	0.75	0.82	43	14	129	28
Joon Yeul Nam	External validation	155	80	75	0.73	0.94	58	22	71	4
Kenta Hamada	Internal validation	68	34	34	0.735	0.794	25	9	27	7
Chang Seok Bang	External validation	206	80	126	0.884	0.897	71	9	113	13
Chang Seok Bang	External validation 2	1597	253	1344	0.685	0.837	173	80	1125	219

SM, Submucosal invasion lesions; M, Mucosal invasion lesions.

#### Model accuracy

**Fig. 3:**
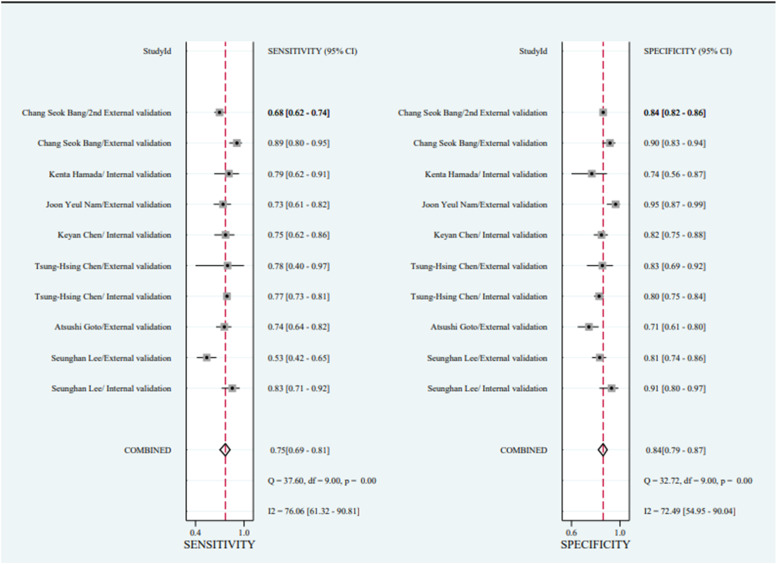
Forest plots of pooled sensitivity (left) and specificity (right) for AI-based prediction of submucosal invasion.

**Fig. 4:**
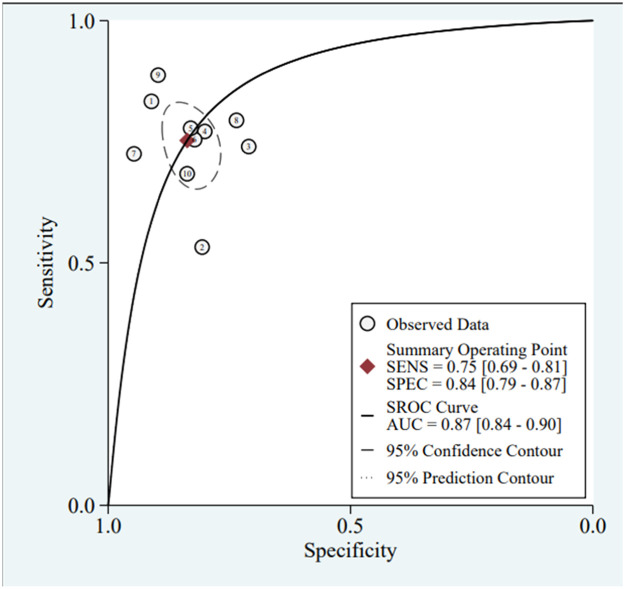
SROC curve of diagnostic performance.

A total of ten validation cohorts were synthesized in the meta-analysis to determine the diagnostic accuracy of AI-driven endoscopic systems for detecting SM invasion in gastric neoplasms. The pooled performance metrics are presented in Fig. 3, showing an overall sensitivity of 0.75 (95% CI 0.69–0.81). Sensitivity estimates displayed high heterogeneity (*I*^2^ = 76.06%, 95% CI 61.32%–90.81%). The corresponding pooled specificity reached 0.84 (95% CI 0.79–0.87), with heterogeneity remaining moderate (*I*^2^ = 72.49%, 95% CI 54.95%–90.04%). The summary ROC curve yielded an AUC of 0.87 (95% CI 0.84–0.90), indicating a strong overall ability of AI models to differentiate lesions with SM invasion from those confined to the mucosa. The ROC curve illustrating this aggregated diagnostic performance is displayed in Fig. 4.

#### Bias and utility analysis

**Fig. 5:**
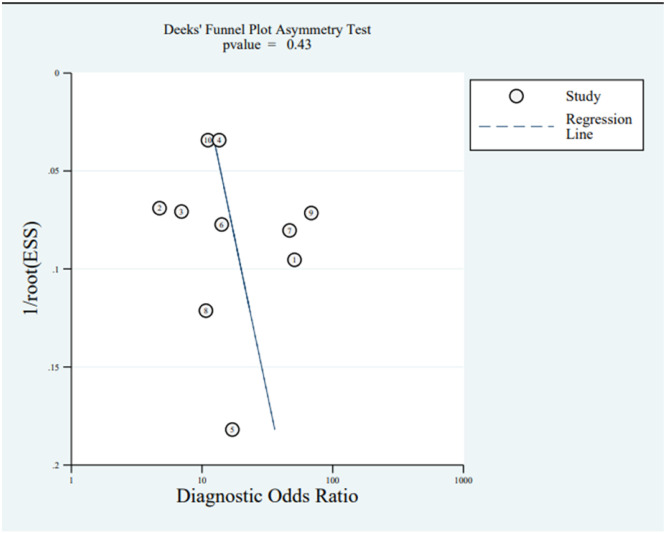
Deeks’ funnel plot assessing publication bias.

**Fig. 6:**
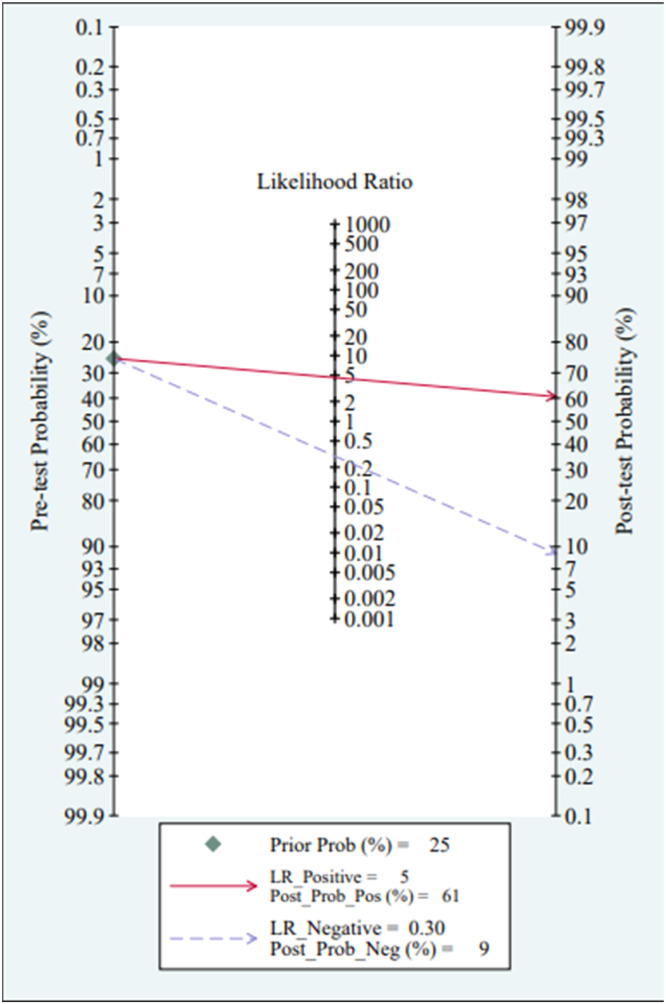
Fagan nomogram showing post-test probabilities.

Assessment of publication bias using Deeks’ funnel plot revealed no statistically significant asymmetry (p = 0.43), suggesting a low likelihood of selective reporting among the included studies. This analysis is presented in Fig. 5. To further appraise the clinical utility of AI-assisted endoscopic models for predicting SM invasion in gastric neoplasms, a Fagan nomogram was constructed. As shown in Fig. 6, applying a pre-test probability of 25% resulted in a post-test probability of 61% following a positive AI prediction, corresponding to a positive likelihood ratio of 5. Conversely, a negative result reduced the post-test probability to 9%, aligned with a negative likelihood ratio of 0.30. Collectively, these values demonstrate that AI-based diagnostic models meaningfully shift disease probability in both directions, underscoring their potential relevance for real-time endoscopic decision-making.

#### Subgroup analysis

Because considerable heterogeneity was observed in both sensitivity and specificity, subgroup analyses were conducted to determine whether differences in study design or methodological features contributed to the observed variability. The analyses focused on the following predefined covariates:

1. Publication period, comparing studies conducted before 2024 with those published in 2024 or later.
2. Type of validation strategy, contrasting internally validated models with those evaluated using external datasets.
3. Geographic region, distinguishing studies originating from Korea from those conducted elsewhere.
4. Operational definition of SM invasion, specifically comparing studies that applied the commonly used criterion of SM1 < 500 µm and SM2 ≥ 500 µm against studies employing alternative depth-of-invasion definitions.
5. Software environment used for model construction, differentiating Python-based workflows from models developed using other analytical platforms.

These subgroup evaluations aimed to determine whether such variations influenced diagnostic performance and may account for part of the substantial heterogeneity observed in the primary pooled estimates. A comprehensive summary of the subgroup-specific diagnostic metrics is provided in Table 3. Because the meta-analysis included only ten validation cohorts overall, and several subgroup strata contained only four to six cohorts (Table 3), these subgroup findings should be considered exploratory and interpreted cautiously.

**Table 3:** Subgroup-specific diagnostic performance.

Covariate		n	Sensitivity	p	Specificity	p
Year of publication	2024	5	0.74 [95% CI: 0.65–0.82]	0.01	0.83 [95% CI: 0.78–0.89]	<0.001
	Before 2024	5	0.77 [95% CI: 0.69–0.84]		0.84 [95% CI: 0.79–0.90]	
Dataset	External validation	6	0.72 [95% CI: 0.65–0.80]	<0.001	0.84 [95% CI: 0.80–0.89]	<0.001
	Internal validation	4	0.79 [95% CI: 0.71–0.86]		0.82 [95% CI: 0.76–0.89]	
Origin	Republic of Korea	5	0.74 [95% CI: 0.67–0.82]	<0.001	0.87 [95% CI: 0.83–0.90]	<0.001
	Other countries	5	0.77 [95% CI: 0.69–0.84]		0.79 [95% CI: 0.74–0.84]	
SM invasion definition	SM1 < 500 µm / SM2 ≥ 500 µm	4	0.73 [95% CI: 0.63–0.82]	0.01	0.80 [95% CI: 0.73–0.87]	<0.001
	Others	6	0.77 [95% CI: 0.70–0.84]		0.85 [95% CI: 0.81–0.89]	
Development Software	Python	6	0.74 [95% CI: 0.66–0.82]	0.01	0.81 [95% CI: 0.77–0.85]	<0.001
	Others	4	0.77 [95% CI: 0.69–0.86]		0.87 [95% CI: 0.82–0.91]	

N, Number; SM, Submucosal,.

### Publication period (pre-2024 vs. 2024 and later)

When stratified by publication year, studies published in 2024 or later had a pooled sensitivity of 0.74 (95% CI 0.65–0.82), slightly lower than the estimate for studies published before 2024 (0.77, 95% CI 0.69–0.84). Specificity followed a similar pattern, with more recent studies reporting a value of 0.83 (95% CI 0.78–0.89), compared with 0.84 (95% CI 0.79–0.90) in earlier publications. Although the absolute differences were modest, both sensitivity (p = 0.01) and specificity (p < 0.001) were statistically significant.

### Validation strategy (external vs. internal)

Model performance also differed depending on the type of validation used. Externally validated systems demonstrated a pooled sensitivity of 0.72 (95% CI 0.65–0.80), which was notably lower than that of models tested exclusively on internal datasets 0.79 (95% CI 0.71–0.86). Meanwhile, specificity values were broadly comparable between the two groups (0.84 [95% CI 0.80–0.89] for external validation vs. 0.82 [95% CI 0.76–0.89] for internal validation). Despite the relative closeness in specificity, both sensitivity and specificity differences were statistically significant (p < 0.001 for each).

### Geographic region (Korea vs. other countries)

Studies originating from the Republic of Korea yielded a pooled sensitivity of 0.74 (95% CI 0.67–0.82), whereas investigations conducted in other regions produced a slightly higher sensitivity of 0.77 (95% CI 0.69–0.84). Specificity showed a more pronounced difference: Korean cohorts achieved 0.87 (95% CI 0.83–0.90), while studies from other countries reported a specificity of 0.79 (95% CI 0.74–0.84). The differences in both metrics were statistically meaningful (p < 0.001).

### Operational definition of SM invasion (SM1/SM2 vs. alternative definitions)

Analyses stratified by the definition of SM invasion revealed that studies applying the commonly accepted SM1 < 500 µm and SM2 ≥ 500 µm criteria reported a pooled sensitivity of 0.73 (95% CI 0.63–0.82). In contrast, studies adopting alternative depth-of-invasion thresholds showed a higher sensitivity of 0.77 (95% CI 0.70–0.84). Specificity estimates followed the same direction, with the SM1/SM2 subgroup yielding 0.80 (95% CI 0.73–0.87), whereas studies using other definitions reached 0.85 (95% CI 0.81–0.89). Both sensitivity (p = 0.01) and specificity (p < 0.001) differences were statistically significant.

### Software environment for model development (Python vs. other platforms)

Performance also varied across computational environments used for model construction. Models implemented in Python exhibited a pooled sensitivity of 0.74 (95% CI 0.66–0.82), slightly lower than the sensitivity observed in studies using alternative platforms 0.77 (95% CI 0.69–0.86). Specificity differences were more pronounced: Python-based systems achieved 0.81 (95% CI 0.77–0.85), compared with 0.87 (95% CI 0.82–0.91) for models developed using other software. Both comparisons showed significant p-values (sensitivity p = 0.01; specificity p < 0.001).

## Discussion

Gastric cancer remains a leading global cause of cancer-related mortality,^26^ underscoring the importance of accurately assessing submucosal invasion in EGC. Because invasion of ≥500 µm markedly increases the risk of lymph node metastasis and often necessitates surgical intervention, reliable depth prediction is essential for appropriate treatment selection. However, conventional WLE and EUS demonstrate only moderate accuracy and are subject to substantial operator variability,^27^ highlighting the need for more objective and reproducible diagnostic approaches. In this context, recent advances in AI offer a promising strategy to enhance consistency and improve the precision of invasion-depth assessment.

In this meta-analysis, AI-based endoscopic systems demonstrated a strong overall ability to distinguish SM invasive lesions from M confined lesions, achieving a pooled sensitivity of 0.75 and specificity of 0.84 across 10 validation cohorts. These findings indicate that current AI algorithms are practical for both detecting deeper invasion and accurately ruling out lesions suitable for endoscopic resection. The summary AUC of 0.87 further confirms the substantial discriminative power of these models, placing their diagnostic performance within the upper range of contemporary tools used for evaluating invasion depth in EGC.

The Fagan nomogram further highlights the clinical utility of AI-assisted assessment. Assuming a pre-test probability of 25%, a positive prediction increases the likelihood of SM invasion to 61%, whereas a negative result decreases it to 9%. These probability shifts are clinically meaningful, as they can influence the choice between proceeding with ESD or recommending surgical resection, particularly in borderline or visually ambiguous cases.

Several meta-analyses in related endoscopic fields provide essential context for interpreting the present findings. Hassan et al.^28^ demonstrated that real-time DL computer-aided detection systems consistently improved colorectal neoplasia detection across randomized trials, highlighting AI’s broader potential to augment endoscopic performance beyond human-only assessment. Similarly, Ma et al.^29^ reported high pooled sensitivity (0.90) and specificity (0.91) for CNNs in detecting early esophageal cancer, underscoring the strong diagnostic capability of CNN-based models in early gastrointestinal malignancy. In the inflammatory bowel disease domain, Jahagirdar et al.^30^ showed that CNN-driven systems achieved excellent accuracy in grading ulcerative colitis severity, with overall accuracy exceeding 90%. Collectively, these findings reveal a consistent pattern across gastrointestinal endoscopy: AI models trained on endoscopic images often deliver robust diagnostic performance that can match or exceed that of traditional methods. The pooled AUC of 0.87 aligns with this broader evidence base, suggesting that AI maintains strong discriminative power even for more complex and nuanced tasks, such as invasion-depth prediction. Agarwal et al.^14^ evaluated AI-assisted endoscopy for both EGC detection and invasion-depth assessment and reported higher pooled performance for depth prediction (sensitivity ≈0.84, specificity ≈0.89; AUC ≈0.89). In contrast, the pooled estimates were lower (sensitivity = 0.75, specificity = 0.84; AUC = 0.87) and therefore should not be described as identical. This difference is likely explained by methodological and clinical scope choices that were intentionally applied in the present study. First, the quantitative synthesis was restricted to validation cohorts only, and the authors deliberately excluded training-set performance to reduce overfitting inflation and preserve generalizability. Second, the subgroup results demonstrate that sensitivity is meaningfully lower under external validation (0.72) compared with internal validation (0.79), consistent with performance attenuation when models are transported across populations, devices, and workflows. Finally, broader depth-assessment meta-analyses may include studies with different endpoint definitions or validation structures that tend to yield higher pooled sensitivity. Accordingly, the pooled results should be interpreted as a more conservative estimate of real-world performance from validation cohorts, reinforcing that AI should be used as an adjunct to comprehensive endoscopic assessment and that future models should prioritize sensitivity and undergo rigorous external, multicenter validation.

Overall, the methodological quality of the included studies was satisfactory, with most demonstrating low risk of bias across QUADAS-2 domains and minimal concerns regarding applicability. Nonetheless, several limitations were identified. One study exhibited a high risk of bias in patient selection due to its retrospective, category-balanced design, which may not reflect the natural distribution of lesions encountered in clinical practice.^23^ Another study had an unclear risk of selection bias because only a single image per patient was manually chosen, potentially reducing dataset representativeness.^22^ Additionally, one investigation showed unclear risk in the index test domain, as endoscopic features were retrospectively interpreted by experienced clinicians who may have been aware of the histologic outcomes.^21^ Despite these isolated concerns, the majority of studies employed sound methodological approaches, supporting the overall reliability and validity of the pooled diagnostic estimates.

Subgroup analyses were performed to explore potential sources of heterogeneity; however, given the limited number of cohorts, the observed differences should be viewed as exploratory. Studies published in 2024 or later showed slightly lower sensitivity and specificity than earlier investigations, a trend that may reflect stricter methodological standards, increased reliance on external validation, and the incorporation of more heterogeneous datasets in recent research. The validation strategy was one of the most influential determinants of performance: models evaluated on external datasets consistently demonstrated reduced sensitivity compared with internally validated systems, underscoring the well-known issue of overfitting in AI research and the performance decrement that occurs when algorithms are applied to new populations, imaging devices, or institutional workflows. Geographic variation also contributed to these differences. Korean studies exhibited higher specificity but slightly lower sensitivity than those conducted elsewhere, suggesting that regional differences in endoscopic training, imaging protocols, equipment characteristics, or underlying disease patterns may affect both the quality of training data and model behavior.

In addition, the included studies differed in lesion spectrum, morphology, and the precise clinical diagnostic task being modeled, which can meaningfully influence diagnostic performance. Some cohorts enrolled broader gastric neoplasms and trained image-based CNN models across a wider range of lesion appearances, whereas others focused specifically on early gastric cancer and the clinically subtle distinction between M and SM1 invasion, where depressed-type or morphologically subtle lesions may be overrepresented. Several studies also differed in their input features and modeling approach (incorporation of structured morphological or colorimetric variables versus endoscopy-image or image-video frameworks), as well as in the stringency of internal versus external validation. Accordingly, the pooled estimates in the present meta-analysis should be interpreted as average performance across related, though not identical, diagnostic scenarios, and performance in a given practice setting may be higher or lower depending on lesion mix, morphology, invasion-depth thresholds, and validation strategy. This is consistent with established observations that diagnostic accuracy metrics can vary across patient and disease spectra.^31^

Also, a recent systematic review by Agarwal et al.^14^ evaluated AI-assisted endoscopy for both EGC detection and invasion-depth assessment. While there is partial overlap in primary studies, the review differs in scope and analytic approach: the authors focused specifically on optical endoscopy-based prediction of SM invasion (binary SM+ vs. mucosa-confined disease), used validation-set data only for quantitative synthesis, and required sufficient reporting to reconstruct 2 × 2 tables. In contrast, broader depth-assessment reviews may include EUS-based AI systems and/or studies using multi-class depth staging that cannot be harmonized to a binary SM invasion endpoint, which can increase the number of “depth” studies counted. Accordingly, the pooled estimates should be interpreted as performance for a clinically actionable SM invasion decision framework derived from validation cohorts, complementing prior broader reviews.

Variability in the operational definition of SM invasion similarly affected pooled accuracy. Studies adopting the commonly used SM1 < 500 µm and SM2 ≥ 500 µm thresholds reported lower sensitivity and specificity than studies applying alternative histopathologic criteria, suggesting that inconsistencies in reference standards may affect label quality and classification boundaries. Finally, the software environment used for model development appeared to contribute to heterogeneity: Python-based workflows yielded slightly lower sensitivity and a more pronounced reduction in specificity compared with models developed on other platforms. Although the underlying reasons are unclear, these differences may stem from variations in preprocessing pipelines, augmentation protocols, default model architectures, or framework-dependent implementation decisions. Collectively, these subgroup findings highlight the multifactorial nature of heterogeneity in AI-driven depth prediction and emphasize the need for greater standardization in imaging protocols, labeling criteria, and model evaluation practices. Among the evaluated factors, validation strategy and geographic origin exerted the most significant influence on diagnostic performance, underscoring the need for externally validated, internationally diverse datasets in future AI research. Compared with prior meta-analyses,^14^ this review is intended as a confirmatory synthesis that emphasizes clinical transportability. First, the authors pooled validation-cohort performance only, excluding training-set results to avoid overfitting-related inflation and provide a more conservative estimate for practice implementation. Second, the authors focused on the clinically actionable endpoint of SM invasion versus mucosa-confined disease, aligning accuracy estimates with treatment selection for ESD versus surgery. Third, the authors conducted predefined subgroup analyses (validation strategy, region, publication period, SM definition, and development environment) to explain heterogeneity and identify conditions under which sensitivity may decrease, particularly under external validation. Finally, the authors translated pooled likelihood ratios into post-test probabilities using a Fagan nomogram to support bedside interpretability.

A key limitation in subgroup analysis is the small evidence base available for quantitative synthesis (ten validation cohorts from seven studies), which constrains the reliability of subgroup comparisons. Several subgroup strata in Table 3 contain only four to six cohorts, which increases the risk of spurious differences, imprecise estimates, and unstable p-values, and precludes robust meta-regression. Accordingly, subgroup results in this review should be interpreted as hypothesis-generating signals about potential sources of heterogeneity and transportability (for example, external vs. internal validation), rather than definitive evidence of performance differences.

In addition, one externally validated cohort was very small and likely to yield unstable accuracy estimates. In particular, the external validation dataset in Tsung-Hsing Chen et al.^20^ included only 56 patients, with just 9 SM-positive cases, making sensitivity or specificity estimates highly sample-dependent and prone to large random error. Such small external cohorts can disproportionately influence pooled estimates and subgroup comparisons, so the external-validation findings should be interpreted cautiously and confirmed in larger multicenter external datasets.

Future research should prioritize the development of prospective, multicenter studies that more accurately reflect real-world clinical practice. Most existing investigations rely on retrospective still-image datasets, which limit ecological validity; therefore, next-generation models should incorporate real-time video analysis, dynamic mucosal patterns, and streaming endoscopic signals. Integrating multimodal inputs, including WLE, magnifying NBI, EUS findings, and patient-level clinical variables, may further enhance predictive performance and better approximate the decision-making process used by experienced endoscopists. Standardization is another critical need. Harmonized definitions of SM invasion, unified labeling protocols, and shared imaging preprocessing pipelines would reduce interstudy variability and improve the reproducibility of future models. Additionally, AI systems should undergo rigorous external validation across diverse geographic regions and device platforms to ensure generalizability, particularly in Western populations that remain underrepresented in the current evidence base.

Beyond technical refinement, future work should also focus on clinical integration. Evaluating how AI-assisted depth prediction influences therapeutic decision-making, ESD outcomes, procedural time, and interobserver variability will be essential for understanding its real-world utility. User-interface optimization, latency reduction, and workflow compatibility are similarly crucial for successful deployment in endoscopy suites. Finally, regulatory frameworks, data privacy considerations, and explainability features will increasingly shape the translation of AI tools from research prototypes into clinically trusted decision-support systems. Addressing these issues will be crucial for realizing the full potential of AI-driven depth prediction in EGC management.

## Conclusion

Artificial intelligence shows strong potential as an adjunct tool for predicting submucosal invasion in gastric neoplasms. Across eight studies in the systematic review and ten validation cohorts from seven studies in the meta-analysis, AI systems demonstrated robust diagnostic performance (sensitivity = 0.75, specificity = 0.84, AUC = 0.87), with clinically meaningful shifts in post-test probability. Although heterogeneity was substantial, reflecting differences in validation strategy, geographic origin, imaging protocols, and histopathologic definitions, AI models consistently improved discrimination between M and SM disease. These findings support AI as a valuable aid for treatment planning in EGC, while highlighting the need for prospective multicenter studies, real-time video analysis, standardized invasion criteria, and rigorous external validation to ensure reliable and generalizable clinical integration.

## Declaration of generative AI and AI-assisted technologies in the manuscript preparation process

During the preparation of this work, the authors used ChatGPT to assist in refining the academic writing style and improving readability. After using this tool/service, the authors reviewed and edited the content as needed and take full responsibility for the content of the published article.

## Ethics approval and consent to participate

Not applicable.

## Data availability

All data extracted and analyzed in this study are available within the article and its Supplementary Materials.

## Funding

This research did not receive any specific grant from funding agencies in the public, commercial, or not-for-profit sectors.

## Declaration of competing interest

The authors declare that they have no known competing financial interests or personal relationships that could have appeared to influence the work reported in this paper.

## Appendix Supplementary materials

Image, application 1

## Footnotes

Supplementary material associated with this article can be found, in the online version, at doi:10.1016/j.clinsp.2026.101010.

## References

[1] Kuroki K., Oka S., Tanaka S., Yorita N., Hata K., Kotachi T. (2021). Preceding endoscopic submucosal dissection in submucosal invasive gastric cancer patients does not impact clinical outcomes. Sci Rep.

[2] Wang X., Wu C., Yue S., Zhou M., Zhuo E., Wu X. (2025). Current status and trends of machine learning applied in clinical research of gastric cancer from 2004 to 2023: global bibliometric and visual analysis. Front Oncol.

[3] Ziogas D.I., Kalakos N., Manolakis A., Voulgaris T., Vezakis I., Tadic M. (2025). Endoscopic ultrasound (EUS) in gastric cancer: current applications and future perspectives. Dis (Basel, Switzerland).

[4] Zhao J., Sun Z., Liang J., Guo S., Huang D. (2021). Endoscopic submucosal dissection for early gastric cancer in elderly vs. Non-Elderly Patients.

[5] Bollschweiler E., Plum P.S. (2024). Prediction of lymphatic invasion for patients with early gastric cancer: a review. AME Clin Trials Rev.

[6] Park W.G., Shaheen N.J., Cohen J., Pike I.M., Adler D.G., Inadomi J.M. (2015). Quality indicators for EGD. Gastrointest Endosc.

[7] Pimenta-Melo A.R., Monteiro-Soares M., Libânio D., Dinis-Ribeiro M. (2016). Missing rate for gastric cancer during upper gastrointestinal endoscopy: a systematic review and meta-analysis. Eur J Gastroenterol Hepatol.

[8] Adler D.G., Bakis G., Coyle W.J., DeGregorio B., Dua K.S., Lee L.S. (2012). Principles of training in GI endoscopy. Gastrointest Endosc.

[9] Wang Y.Y., Liu B., Wang J.H. (2025). Application of deep learning-based convolutional neural networks in gastrointestinal disease endoscopic examination. World J Gastroenterol.

[10] Lei C., Sun W., Wang K., Weng R., Kan X., Li R. (2025). Artificial intelligence-assisted diagnosis of early gastric cancer: present practice and future prospects. Ann Med.

[11] Huang C., Song Y., Dong J., Yang F., Guo J., Sun S. (2025). Diagnostic performance of AI-assisted endoscopy diagnosis of digestive system tumors: an umbrella review. Front Oncol.

[12] Gong E.J., Woo J., Lee J.J., Bang CS. (2025). Role of artificial intelligence in gastric diseases. World J Gastroenterol.

[13] Matsubayashi C.O., Cheng S., Hulchafo I., Zhang Y., Tada T., Buxbaum J.L. (2024). Artificial intelligence for gastric cancer in endoscopy: from diagnostic reasoning to market. Dig Liver Dis.

[14] Agarwal S., Rajput M.S., Pandey S., Ramai D., Tabibian J.H., Ofosu A. (2025). Evaluation of the use of convoluted neural network for detecting early gastric cancer and predicting its invasion depth: a systematic review and meta-analysis. Dig Liver Dis.

[15] McInnes M.D.F., Moher D., Thombs B.D., McGrath T.A., Bossuyt P.M., Clifford T. (2018). Preferred reporting items for a systematic review and meta-analysis of diagnostic test accuracy studies: the PRISMA-DTA Statement. JAMA.

[16] Ouzzani M., Hammady H., Fedorowicz Z., Elmagarmid A. (2016). Rayyan ‒ a web and mobile app for systematic reviews. Syst Rev.

[17] Whiting P.F., Rutjes A.W.S., Westwood M.E., Mallett S., Deeks J.J., Reitsma J.B. (2011). QUADAS-2: a revised tool for the quality assessment of diagnostic accuracy studies. Ann Intern Med.

[18] Lee S., Jeon J., Park J., Chang Y.H., Shin C.M., Oh M.J. (2024). An artificial intelligence system for comprehensive pathologic outcome prediction in early gastric cancer through endoscopic image analysis (with video). Gastric Cancer Off J Int Gastric Cancer Assoc Japanese Gastric Cancer Assoc.

[19] Goto A., Kubota N., Nishikawa J., Ogawa R., Hamabe K., Hashimoto S. (2023). Cooperation between artificial intelligence and endoscopists for diagnosing invasion depth of early gastric cancer. Gastric Cancer Off J Int Gastric Cancer Assoc Japanese Gastric Cancer Assoc.

[20] Chen T.H., Kuo C.F., Lee C., Yeh T.S., Lan J., Huang S.C. (2024). Artificial intelligence model for a distinction between early-stage gastric cancer invasive depth T1a and T1b. J Cancer.

[21] Chen K., Wang Y., Lang Y., Yang L., Guo Z., Wu W. (2024). Machine learning models to predict submucosal invasion in early gastric cancer based on endoscopy features and standardized color metrics. Sci Rep.

[22] Nam J.Y., Chung H.J., Choi K.S., Lee H., Kim T.J., Soh H. (2022). Deep learning model for diagnosing gastric mucosal lesions using endoscopic images: development, validation, and method comparison. Gastrointest Endosc.

[23] Hamada K., Kawahara Y., Tanimoto T., Ohto A., Toda A., Aida T. (2022). Application of convolutional neural networks for evaluating the depth of invasion of early gastric cancer based on endoscopic images. J Gastroenterol Hepatol.

[24] Bang C.S., Lim H., Jeong H.M., Hwang S.H. (2021). Use of endoscopic images in the prediction of submucosal invasion of gastric neoplasms: automated deep learning model development and usability study. J Med Internet Res.

[25] Zhu Y., Wang Q.C., Xu M.D., Zhang Z., Cheng J., Zhong Y.S. (2019). Application of convolutional neural network in the diagnosis of the invasion depth of gastric cancer based on conventional endoscopy. Gastrointest Endosc.

[26] Sung H., Ferlay J., Siegel R.L., Laversanne M., Soerjomataram I., Jemal A. (2021). Global cancer statistics 2020: GLOBOCAN estimates of incidence and mortality worldwide for 36 cancers in 185 countries. CA Cancer J Clin.

[27] Li X., Zhu M., Wang Y., Niu Y., Ji M., Li P. (2021). Diagnostic efficacy and decision-making role of preoperative endoscopic ultrasonography in early gastric cancer. Front Med.

[28] Hassan C., Spadaccini M., Iannone A., Maselli R., Jovani M., Chandrasekar V.T. (2021). Performance of artificial intelligence in colonoscopy for adenoma and polyp detection: a systematic review and meta-analysis. Gastrointest Endosc.

[29] Ma H., Wang L., Chen Y., Tian L. (2022). Convolutional neural network-based artificial intelligence for the diagnosis of early esophageal cancer based on endoscopic images: a meta-analysis. Saudi J Gastroenterol Off J Saudi Gastroenterol Assoc.

[30] Jahagirdar V., Bapaye J., Chandan S., Ponnada S., Kochhar G.S., Navaneethan U. (2023). Diagnostic accuracy of convolutional neural network-based machine learning algorithms in endoscopic severity prediction of ulcerative colitis: a systematic review and meta-analysis. Gastrointest Endosc.

[31] Ransohoff D.F., Feinstein A.R. (1978). Problems of spectrum and bias in evaluating the efficacy of diagnostic tests. N Engl J Med.

